# Factors affecting the changes in antihypertensive medications in patients with hypertension

**DOI:** 10.3389/fcvm.2022.999548

**Published:** 2022-09-30

**Authors:** Tae Kyu Chung, Yoomin Jeon, YeSol Hong, Suyeon Hong, Jun Sik Moon, Howard Lee

**Affiliations:** ^1^Department of Applied Bioengineering, Graduate School of Convergence Science and Technology, Seoul National University, Seoul, South Korea; ^2^Center for Convergence Approaches in Drug Development, Seoul, South Korea; ^3^Department of Molecular Medicine and Biopharmaceutical Sciences, Graduate School of Convergence Science and Technology, Seoul National University, Seoul, South Korea; ^4^Department of Clinical Pharmacology and Therapeutics, Seoul National University College of Medicine and Hospital, Seoul, South Korea; ^5^Advanced Institutes of Convergence Technology, Suwon, South Korea

**Keywords:** epidemiology, hypertension, common data model, anti-hypertensive medication, cardiovascular disease

## Abstract

As frequent changes in anti-hypertensive (HTN) medications may reduce adherence to the treatments, identifying modifiable factors leading to changes in anti-HTN medications can help clinicians optimize treatment strategies for individual patients. We performed this study to explore the pattern of anti-HTN medications and to identify factors that are associated with the changes in anti-HTN medications. To this end, we used a clinical database of Seoul National University Hospital, extracted, transformed, and loaded by the observational medical outcomes partnership common data model. Demographic and all recorded clinical diagnoses, medications, and procedures data of eligible subjects were collected. Of 636 subjects who were eligible for this study, 297 subjects with a record of ≥1 anti-HTN medication changes and other 297 subjects without a record of medication change were selected for the study population. High diastolic blood pressure (adjusted odds ratio [OR]: 1.02, 95% confidence interval [CI]: 1.001–1.040, *p* = 0.040), arrhythmia (adjusted OR: 10.01, 95% CI: 1.86–185.57, *p* = 0.030), and angina pectoris with antianginal agents (adjusted OR: 4.85, CI: 1.05–23.89, *p* = 0.046) were associated with the changes in anti-HTN medications, indicating that any patients with these covariates require additional attention to reduce the likelihood of changing anti-HTN medications.

## Introduction

Hypertension (HTN) is a major risk factor for cardiovascular diseases (1). The number of patients with HTN has almost doubled globally in the last 40 years from 594 million (1975) to 1.13 billion (2015), largely driven by aging of the population, obesity, and a higher level of stress (2–4). Although many anti-HTN medications are available, not all patients receive full potential benefits from the treatment (5).

Suboptimal adherence to the anti-HTN medications is a major reason for the reduced benefit of the treatment (6). Frequent changes in anti-HTN medications may reduce adherence to anti-HTN medications (7, 8). Although physicians are likely to change anti-HTN medications to optimize the benefit of the treatment, patients may intentionally stop adhering to their treatment because of frequent changes (9, 10). Furthermore, adherence can be further reduced when the dosage regimen becomes more complex and/or patients experience unanticipated adverse events (7).

Therefore, it is recommended to minimize changes in anti-HTN medications to maximize the benefit of the treatment by increasing adherence. In this regard, identifying factors that can potentially lead to changes in anti-HTN medications may help clinicians optimize treatment strategies for individual patients.

Factors that are associated with anti-HTN medication changes have not been fully evaluated (11–13). Previous studies reported that age, male sex, and visit type (e.g., new vs. follow-up visit) were significantly associated with the changes in anti-HTN medications (12). However, other clinically important covariates such as comorbidity, concomitant medications, laboratory test results, and procedure information have not been adjusted for in their analyses (11, 12).

The objectives of our study were (a) to explore the pattern of anti-HTN medications, and (b) to identify factors that are associated with the changes in anti-HTN medications. To this end, we tested a hypothesis that patients with certain medical conditions are significantly associated with the changes in anti-HTN medications.

## Materials and methods

### Data source

We used a clinical database of Seoul National University Hospital (SNUH), Seoul, South Korea, extracted, transformed, and loaded using by the observational medical outcomes partnership common data model (OMOP CDM, version 5.3.1). The OMOP CDM of SNUH was screened for the period of January 2004 to December 2020. SNUH is a university-affiliated, tertiary-care hospital, the OMOP CDM of which contains 2.3 billion medical records from 3 million Korean patients (14). This study was reviewed and approved by the SNUH Institutional Review Board with obtaining the informed consent waived (IRB No: 2103-140-1206).

### Study subjects

Eligible subjects were those who had been diagnosed with HTN and received ≥1 anti-HTN medications. The index date was defined as the first date of prescription for anti-HTN medication with ≥365 days of prior clinical history identified. On the other hand, the cohort exit date was defined as 1,095 days after the index date (15). We excluded subjects if they had not been continuously treated with anti-HTN medications, where continuous treatment was defined as having prescription records for anti-HTN medications for ≥875 days (i.e., 80% of 1,095 days) without >30 days of drug holiday between any two adjacent prescriptions. Subjects were also excluded if they were younger than 18 years old, had a medical history of major surgery, pregnancy, or a human chorionic gonadotropin >5 mIU/mL between the index date and cohort exit date. Additionally, subjects whose blood pressure measurements were unavailable before the index date were excluded.

Eligible subjects were classified into two groups: the changed and unchanged groups. Subjects were defined as the changed group if ≥1 anti-HTN medication that were not part of their initial regimens have been included, removed, or changed in their prescription records for ≥30 days between the index date and cohort exit date. In contrast, subjects without a record of medication change were defined as the unchanged group. To adjust for the potential differences in index year, age, sex, prescription records, and general medical history between the changed and unchanged groups, 1:1 propensity score matching was performed. After propensity matching, we compared the baseline characteristics of the changed and unchanged groups, which included index year, age, sex, prescription records, lab measurements, surgical procedures, hospitalization, numbers of comorbidities and concomitant medications, and medical histories of general and cardiovascular diseases. Baseline was defined as the period between the index date and a year before the index date. Dyslipidemia, liver disease, and renal disease were operationally defined using the following criteria. Dyslipidemia was total cholesterol ≥240 mg/dL, high density lipoprotein (HDL) <40 mg/dL, low density lipoprotein (LDL) ≥160 mg/dL, triglyceride (TG) ≥200 mg/dL, or having any diagnosis of dyslipidemia-related diseases (16). Likewise, liver disease was aspartate aminotransferase (AST) or alanine transaminase (ALT) ≥120 IU/L (3 times the upper limit of normal) or having any diagnosis of steatosis of liver, cirrhosis, or hepatitis. Furthermore, renal disease was serum creatinine >1.4 mg/dL, estimated glomerular filtration rate (eGFR) <60 ml/min/1.73 m^2^ using the modification of diet in renal disease (MDRD) equation, or having any diagnosis of renal failure or chronic kidney disease (17–19).

### Anti-hypertensive medications

We categorized anti-HTN medications into the following six classes: angiotensin-converting-enzyme inhibitors (ACEi), angiotensin receptor blockers (ARB), beta-blockers (BB), calcium channel blockers (CCB), diuretics (DU), and others (e.g., alpha-blockers and vasodilators) (20). A change in anti-HTN medications was defined as one of the following four situations: (1) when ≥1 anti-HTN medications were added or changed from the previous monotherapy; (2) when ≥1 anti-HTN medications were added, removed, or changed from the previous combination therapies; (3) when the dose of ≥1 anti-HTN medications were changed; or (4) any combination of 1–3. We assumed a subject was on combination therapy if there were ≥2 active ingredients or anti-HTN medication classes over the same timeframe.

### Exploration of the patterns of anti-HTN medications

The patterns of anti-HTN medications were characterized by anti-HTN medication classes. To this end, a Sankey plot was generated using networkD3 package in R version 3.5.1 (R Foundation, Vienna, Austria) to visualize the use of anti-HTN medications over time (21). We also identified the number of changes in anti-HTN medications by different type of changes, i.e., dose increased, dose reduced, medication added, medication removed, medication changed within the same class, and medication changed to different class.

### Statistical analysis

Continuous variables were described using mean ± standard deviation (SD). Categorical variables were presented as proportion (%). Continuous and categorical variables were compared between the changed and unchanged groups by using Student's *t*-tests and Pearson χ^2^ tests or Fisher's exact tests, respectively.

The association between a covariate and change in anti-HTN medications was assessed using univariate and multivariate logistic regression analyses. The logistic regression models included the main effects and all of the 2nd order interactions between the main effects. To select variables for multivariate logistic regression analyses, stepAIC function from the MASS package in R was used. The stepAIC function selects variables from the initial logistic regression model by identifying the model with the lowest Akaike's An Information Criterion (22). We also conducted a sensitivity analysis to check for the robustness of the results of the main analysis by changing baseline to a period between the index date and 6 months before the index date. The results of the univariate and multivariate logistic regression analyses were reported using an odds ratio (OR) and its 95% confidence intervals (CIs). A *p*-value < 0.05 was considered statistically significant.

## Results

### Subjects

**Figure 1:**
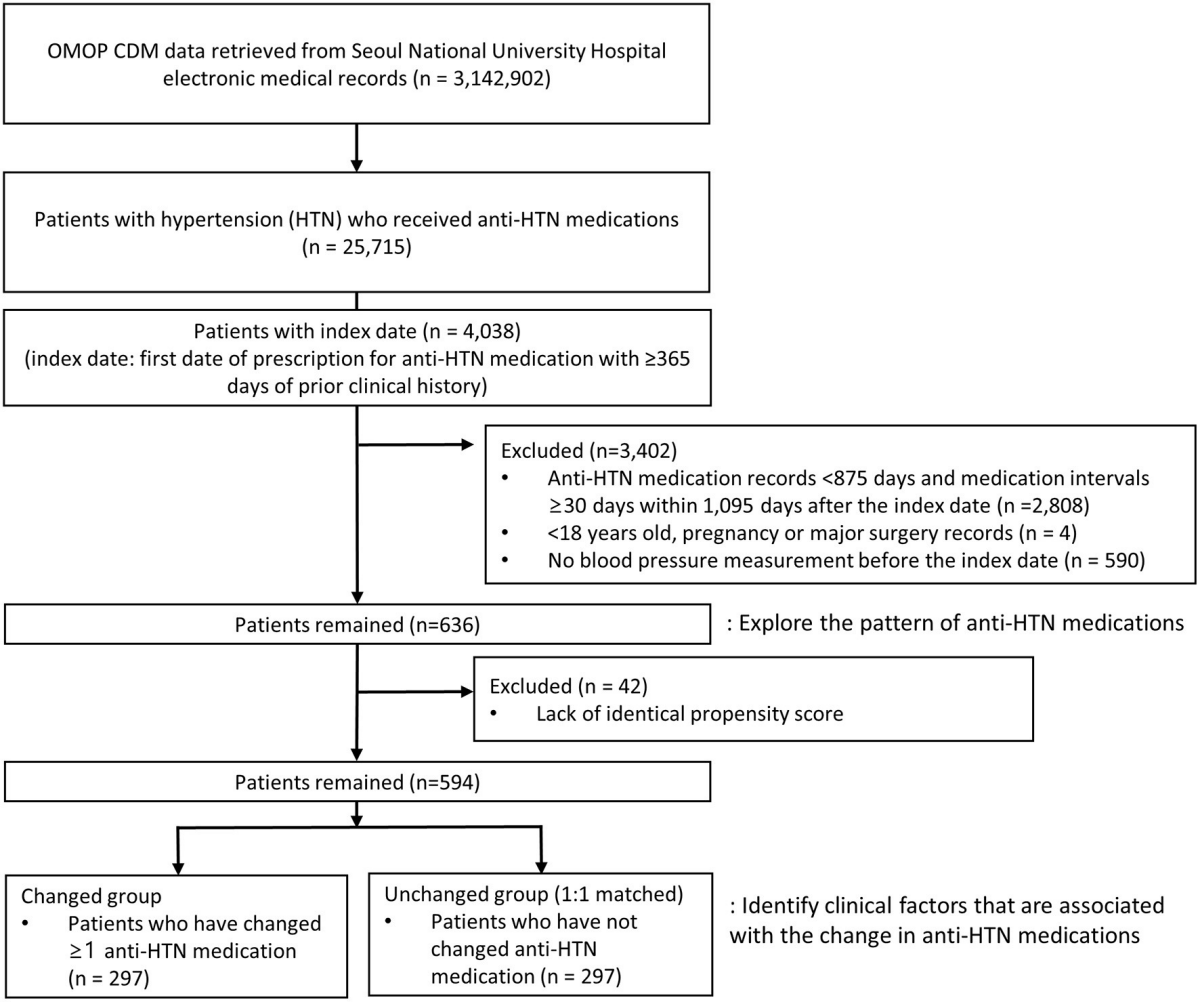
Flow chart of selecting study subjects. OMOP CDM, the observational medical outcomes partnership common data model.

A total of 25,715 subjects with anti-HTN medications were identified, of whom 636 were eligible for this study (Figure 1). After 1:1 propensity score matching, a total of 594 subjects were selected for the study population, 297 of whom fell into the changed and unchanged groups, respectively. All baseline characteristics of subjects in the changed and unchanged groups became comparable and balanced after propensity score matching (Table 1 and Supplementary Figures 1, 2). The mean age was 62.5 years, and slightly over the half of subjects were males (55.2%). Before the index date, 46.1, 21.5, 14.6% of subjects had dyslipidemia, diabetes mellitus, and gastrointestinal diseases, respectively (Table 1). Between the index date and cohort exit date, the proportions of subjects of three diseases have increased by 37.4, 15.7, and 27.3% points (Supplementary Table 1).

**Table 1:** Baseline characteristics.

**Factors**	**Before propensity score matching**				**After propensity score matching**			
	**Total (*n* = 636)**	**Anti-HTN medication changed (*n* = 334)**	**Anti-HTN medication unchanged (*n* = 302)**	***p*-value**	**Total (*n* = 594)**	**Anti-HTN medication changed (*n* = 297)**	**Anti-HTN medication unchanged (n = 297)**	***p*-value**
**Index year**, year ± SD	2012.6 ± 2.0	2012.5 ± 2.0	2012.8 ± 2.0	0.054	2012.6 ± 2.0	2012.7 ± 2.0	2012.5 ± 2.0	0.175
**Demographics**								
Age, year ± SD	62.8 ± 10.8	64.0 ± 11.2	61.9 ± 10.4	0.036	62.5 ± 10.8	62.0 ± 10.3	63.1 ± 11.2	0.247
Male, *n* (%)	353 (55.5)	192 (57.5)	161 (53.3)	0.328	328 (55.2)	169 (56.9)	159 (53.5)	0.458
**Medical history: general**, *n* (%)								
Diabetes mellitus	136 (21.4)	68 (20.4)	68 (22.5)	0.572	128 (21.5)	61 (20.5)	67 (22.6)	0.618
Diabetic neuropathy	5 (0.8)	2 (0.6)	3 (1.0)	0.672	5 (0.8)	2 (0.7)	3 (1.0)	1.000
Dyslipidemia	292 (45.9)	150 (44.9)	142 (47.0)	0.650	274 (46.1)	134 (45.1)	140 (47.1)	0.681
Eye diseases	56 (8.8)	26 (7.8)	30 (9.9)	0.415	53 (8.9)	24 (8.1)	29 (9.8)	0.565
Gastrointestinal diseases	103 (16.2)	62 (18.6)	41 (13.6)	0.110	87 (14.6)	48 (16.2)	39 (13.1)	0.353
Hyperuricemia	11(1.7)	8 (2.4)	3 (1.0)	0.294	8 (1.3)	5 (1.7)	3 (1.0)	0.725
Hypothyroidism	11 (1.7)	8 (2.4)	3 (1.0)	0.294	8 (1.3)	5 (1.7)	3 (1.0)	0.725
Insomnia	13 (2.0)	3 (0.9)	10 (3.3)	0.032	12 (2.0)	3 (1.0)	9 (3.0)	0.080
Liver diseases	81 (12.7)	47 (14.1)	34 (11.3)	0.345	73 (12.3)	39 (13.1)	34 (11.4)	0.617
Mental and behavioral diseases	36 (5.7)	26 (7.8)	10 (3.3)	0.023	23 (3.9)	14 (4.7)	9 (3.0)	0.395
Musculoskeletal diseases	46 (7.2)	25 (7.5)	21 (7.0)	0.916	45 (7.6)	25 (8.4)	20 (6.7)	0.535
Neoplasm	51 (8.0)	28 (8.4)	23 (7.6)	0.834	46 (7.7)	23 (7.7)	23 (7.7)	1.000
Nervous system disorders	17 (2.7)	14 (4.2)	3 (1.0)	0.024	5 (0.8)	2 (0.7)	3 (1.0)	1.000
Obesity	12 (1.9)	6 (1.8)	6 (2.0)	1.000	10 (1.7)	4 (1.3)	6 (2.0)	0.750
Prediabetes	22 (3.5)	11 (3.3)	11 (3.6)	0.981	20 (3.4)	10 (3.4)	10 (3.4)	1.000
Prostatic hyperplasia	19 (3.0)	12 (3.6)	7 (2.3)	0.478	15 (2.5)	8 (2.7)	7 (2.4)	1.000
Pulmonary diseases	31 (4.9)	16 (4.8)	15 (5.0)	1.000	29 (4.9)	14 (4.7)	15 (5.1)	1.000
Renal diseases	65 (10.2)	34 (10.2)	31 (10.3)	1.000	59 (9.9)	28 (9.4)	31 (10.4)	0.784
Symptoms	174 (27.4)	102 (30.5)	72 (23.8)	0.071	155 (26.1)	84 (28.3)	71 (23.9)	0.262
**Medication use**, n (%)								
Analgesic agents	47 (7.4)	30 (9.0)	17 (5.6)	0.144	39 (6.6)	22 (7.4)	17 (5.7)	0.508
Antianginal agents	60 (9.4)	35 (10.5)	25 (8.3)	0.417	51 (8.6)	27 (9.1)	24 (8.1)	0.770
Antianxiety agents	23 (3.6)	12 (3.6)	11 (3.6)	1.000	20 (3.4)	10 (3.4)	10 (3.4)	1.000
Antibacterials	22 (3.5)	13 (3.9)	9 (3.0)	0.681	18 (3.0)	9 (3.0)	9 (3.0)	1.000
Anticoagulant agents	126 (19.8)	77 (23.1)	49 (16.2)	0.040	106 (17.8)	59 (19.9)	47 (15.8)	0.238
Anticonvulsants	9 (1.4)	4 (1.2)	5 (1.7)	0.742	8 (1.3)	4 (1.3)	4 (1.3)	1.000
Antidepressants	12 (1.9)	9 (2.7)	3 (1.0)	0.200	8 (1.3)	5 (1.7)	3 (1.0)	0.725
Antidiabetic agents	103 (16.2)	52 (15.6)	51 (16.9)	0.732	95 (16.0)	45 (15.2)	50 (16.8)	0.654
Antigout agents	10 (1.6)	6 (1.8)	4 (1.3)	0.755	8 (1.3)	5 (1.7)	3 (1.0)	0.725
Gastrointestinal agents	299 (47.0)	162 (48.5)	137 (45.4)	0.476	276 (46.5)	141 (47.5)	135 (45.5)	0.681
HMG-CoA reductase inhibitors	290 (45.6)	140 (41.9)	150 (49.7)	0.060	278 (46.8)	130 (43.8)	148 (49.8)	0.162
Micturition disorder drugs	16 (2.5)	9 (2.7)	7 (2.3)	0.961	15 (2.5)	8 (2.7)	7 (2.4)	1.000
NSAIDs	238 (37.4)	126 (37.7)	112 (37.1)	0.933	222 (37.4)	110 (37.0)	112 (37.7)	0.932
Respiratory system agents	45 (7.1)	27 (8.1)	18 (6.0)	0.374	41 (6.9)	23 (7.7)	18 (6.1)	0.517
Sedatives	157 (24.7)	79 (23.7)	78 (25.8)	0.587	155 (26.1)	78 (26.3)	77 (25.9)	1.000

SD, standard deviation; HTN, hypertension; NSAIDS, non-steroidal anti-inflammatory drugs.

The two sample t-test and χ^2^ test were performed for continuous and categorical variables, respectively.

### Patterns of anti-HTN medications

**Figure 2:**
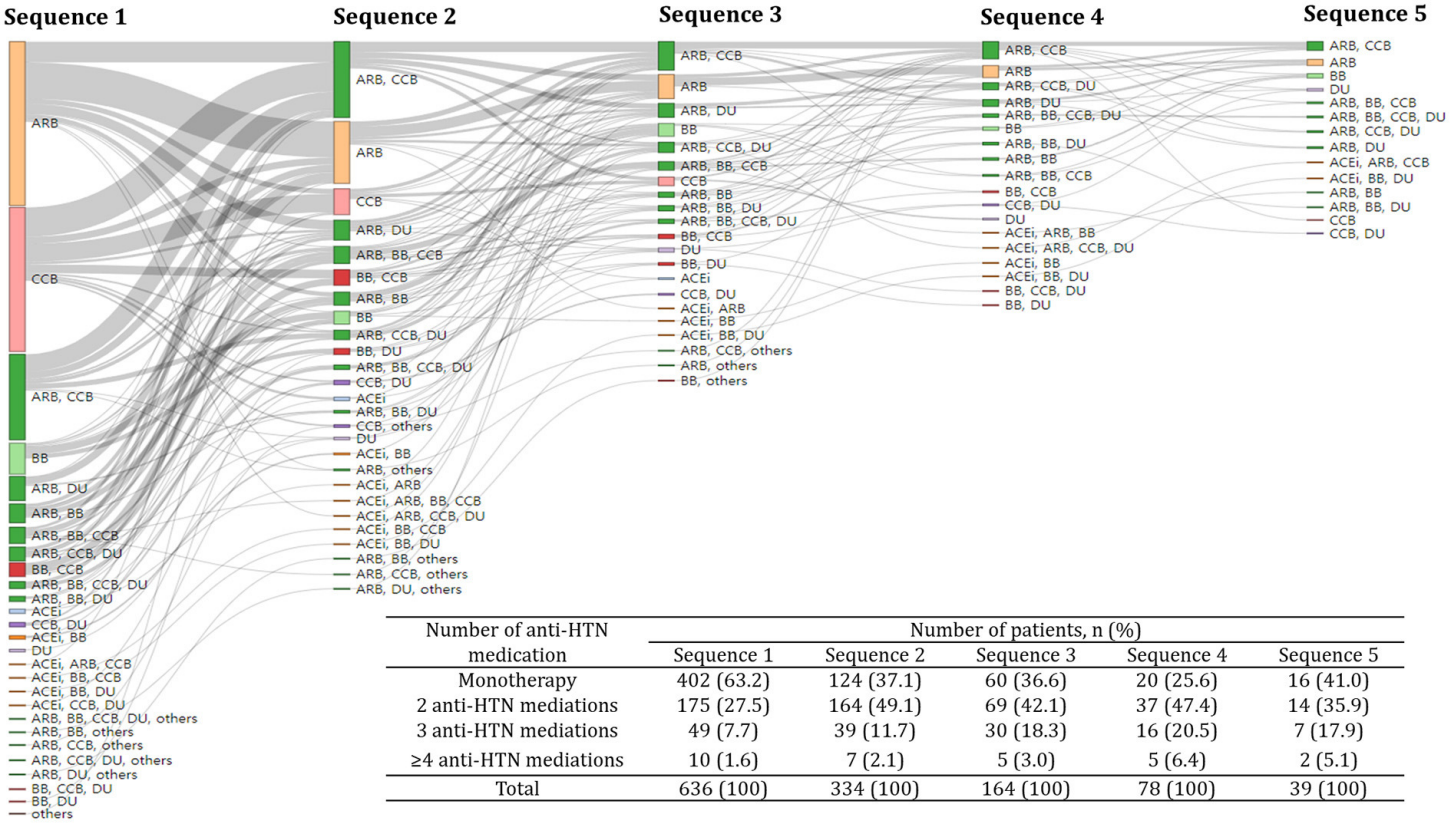
Treatment pathway of anti-hypertensive medications. A Sankey plot showed up to the fifth sequence of anti-hypertensive medication classes. The same color was assigned to the same anti-hypertensive medication class and the size of each colored bar is proportional to the number of subjects who received a particular anti-hypertensive medication class. The inlet table shows the number of subjects who received different number of anti-hypertensive medications for each sequence. HTN, hypertension; ACEi, Angiotensin-converting enzyme inhibitors; ARB, angiotensin receptor blockers; BB, beta-blockers; CCB, calcium channel blockers; DU, diuretics; others, alpha-blockers and vasodilators.

The frequency of changes in anti-HTN medications ranged from 0 to 7, with a median of 2. About two-thirds (63.2%) and one-fourth (27.5%) of subjects received one and two anti-HTN medications, respectively, as the initial treatment (Figure 2). A smaller proportion of subjects (9.6%) received ≥3 anti-HTN medications for the initial anti-HTN treatment. Subjects received ARB (29.9%) most frequently for the first anti-HTN medication, followed by CCB (26.3%), ARB+CCB (15.6%), and BB (5.7%). This pattern was consistently observed in all age groups and in every year between 2010 and 2018 (Table 2). Initial anti-HTN medication choice differed by comorbidities (Table 2). For example, 48.5% of the subjects with diabetes mellitus received ARB for the first anti-HTN medication, while 14.3% of subjects with arrhythmia received BB and 22.2% of subjects with angina pectoris received CCB.

**Table 2:** Initial prescription of anti-hypertensive medications by age, sex, comorbidities, provider's specialty, and year.

**Factors**	**Monotherapy, number of subjects (%)**					**Combination therapy, number of subjects (%)**					**Total, number of subjects**
	**ARB**	**CCB**	**BB**	**ACEi**	**DU**	**ARB+CCB**	**ARB+DU**	**ARB+BB**	**ARB+BB+CCB**	**Other combination therapies**	
First anti-HTN medication	190 (29.9)	167 (26.3)	36 (5.7)	5 (0.8)	3 (0.5)	99 (15.6)	28 (4.4)	22 (3.5)	19 (3.0)	67 (10.5)	636
**Age**											
20–29	1 (25.0)	2 (50.0)	0 (0.0)	0 (0.0)	0 (0.0)	1 (25.0)	0 (0.0)	0 (0.0)	0 (0.0)	0 (0.0)	4
30–39	1 (14.3)	1 (14.3)	0 (0.0)	0 (0.0)	0 (0.0)	2 (28.6)	0 (0.0)	0 (0.0)	1 (14.3)	2 (28.6)	7
40–49	25 (41.0)	17 (27.9)	2 (3.3)	0 (0.0)	0 (0.0)	10 (16.4)	4 (6.6)	0 (0.0)	1 (1.6)	2 (3.3)	61
50–59	49 (30.4)	43 (26.7)	9 (5.6)	2 (1.2)	1 (0.6)	30 (18.6)	7 (4.3)	3 (1.9)	4 (2.5)	13 (8.1)	161
60–69	70 (32.7)	59 (27.6)	12 (5.6)	2 (0.9)	1 (0.5)	31 (14.5)	9 (4.2)	10 (4.7)	4 (1.9)	16 (7.5)	214
70–79	39 (24.4)	39 (24.4)	11 (6.9)	1 (0.6)	1 (0.6)	19 (11.9)	8 (5.0)	7 (4.4)	8 (5.0)	27 (16.9)	160
≥80	5 (17.2)	6 (20.7)	2 (6.9)	0 (0.0)	0 (0.0)	6 (20.7)	0 (0.0)	2 (6.9)	1 (3.4)	7 (24.1)	29
**Sex**											
Male	113 (32.0)	90 (25.5)	18 (5.1)	3 (0.8)	2 (0.6)	63 (17.8)	8 (2.3)	12 (3.4)	6 (1.7)	38 (10.7)	353
Female	77 (27.2)	77 (27.2)	18 (6.4)	2 (0.7)	1 (0.4)	36 (12.7)	20 (7.1)	10 (3.5)	13 (4.6)	29 (10.2)	283
**Comorbidities**											
Diabetes mellitus	66 (48.5)	17 (12.5)	4 (2.9)	0 (0.0)	0 (0.0)	25 (18.4)	5 (3.7)	3 (2.2)	4 (2.9)	12 (8.8)	136
Dyslipidemia	98 (33.6)	74 (25.3)	14 (4.8)	4 (1.4)	2 (0.7)	43 (14.7)	15 (5.1)	8 (2.7)	7 (2.4)	27 (9.2)	292
Liver diseases	31 (38.3)	15 (18.5)	5 (6.2)	0 (0.0)	0 (0.0)	19 (23.5)	3 (3.7)	2 (2.5)	0 (0.0)	6 (7.4)	81
Mental and behavioral diseases	12 (33.3)	8 (22.2)	4 (11.1)	0 (0.0)	0 (0.0)	3 (8.3)	0 (0.0)	2 (5.6)	4 (11.1)	3 (8.3)	36
Neoplasm	16 (31.4)	16 (31.4)	2 (3.9)	0 (0.0)	1 (2.0)	6 (11.8)	1 (2.0)	2 (3.9)	2 (3.9)	5 (9.8)	51
Renal diseases	15 (23.1)	16 (24.6)	6 (9.2)	2 (3.1)	0 (0.0)	10 (15.4)	3 (4.6)	4 (6.2)	1 (1.5)	8 (12.3)	65
Abnormal ECG	5 (21.7)	5 (21.7)	1 (4.3)	0 (0.0)	0 (0.0)	3 (15.4)	1 (4.3)	2 (8.7)	1 (4.3)	5 (21.7)	23
Atrial fibrillation	3 (15.8)	4 (21.1)	4 (21.1)	0 (0.0)	1 (5.3)	0 (0.0)	0 (0.0)	1 (5.3)	2 (10.5)	4 (21.1)	19
Angina pectoris	6 (13.3)	10 (22.2)	3 (6.7)	0 (0.0)	0 (0.0)	5 (11.1)	2 (4.4)	6 (13.3)	1 (2.2)	12 (26.7)	45
Arrhythmia	0 (0.0)	1 (7.1)	2 (14.3)	1 (7.1)	0 (0.0)	0 (0.0)	1 (7.1)	1 (7.1)	1 (7.1)	7 (50.0)	14
Arteriosclerosis	0 (0.0)	0 (0.0)	1 (25.0)	0 (0.0)	0 (0.0)	3 (75.0)	0 (0.0)	0 (0.0)	0 (0.0)	0 (0.0)	4
Cardiomyopathy	0 (0.0)	0 (0.0)	0 (0.0)	0 (0.0)	0 (0.0)	0 (0.0)	1 (25.0)	1 (25.0)	0 (0.0)	2 (50.0)	4
Cerebrovascular disease	33 (40.7)	22 (27.2)	7 (8.6)	3 (3.7)	0 (0.0)	9 (11.1)	4 (4.9)	0 (0.0)	0 (0.0)	3 (3.7)	81
Coronary arteriosclerosis	14 (27.5)	11 (21.6)	4 (7.8)	0 (0.0)	0 (0.0)	10 (19.6)	2 (3.9)	2 (3.9)	2 (3.9)	6 (11.8)	51
Heart failure	2 (20.0)	2 (20.0)	1 (10.0)	0 (0.0)	0 (0.0)	1 (10.0)	0 (0.0)	0 (0.0)	1 (10.0)	3 (30.0)	10
**Provider's specialty**											
Cardiology	27 (22.1)	34 (27.9)	10 (8.2)	3 (2.5)	1 (0.8)	28 (23.0)	8 (6.6)	2 (1.6)	1 (0.8)	8 (6.6)	122
Endocrinology	38 (62.3)	9 (14.8)	0 (0.0)	0 (0.0)	1 (1.6)	10 (16.4)	2 (3.3)	0 (0.0)	0 (0.0)	1 (1.6)	61
Neurology	16 (33.3)	15 (31.3)	4 (8.3)	2 (4.2)	0 (0.0)	10 (20.8)	1 (2.1)	0 (0.0)	0 (0.0)	0 (0.0)	48
**Year**											
2010	24 (32.9)	17 (23.3)	5 (6.8)	1 (1.4)	0 (0.0)	7 (9.6)	3 (4.1)	4 (5.5)	4 (5.5)	8 (11.0)	73
2011	30 (27.8)	28 (25.9)	5 (4.6)	1 (0.9)	1 (0.9)	19 (17.6)	9 (8.3)	6 (5.6)	1 (0.9)	8 (7.4)	108
2012	37 (31.4)	33 (28.0)	3 (2.5)	1 (0.8)	0 (0.0)	20 (16.9)	5 (4.2)	5 (4.2)	4 (3.4)	10 (8.5)	118
2013	31 (30.1)	23 (22.3)	7 (6.8)	0 (0.0)	0 (0.0)	15 (14.6)	4 (3.9)	2 (1.9)	6 (5.8)	15 (14.6)	103
2014	19 (28.8)	20 (30.3)	7 (10.6)	1 (1.5)	1 (1.5)	10 (15.2)	2 (3.0)	2 (3.0)	0 (0.0)	4 (6.1)	66
2015	16 (26.7)	21 (35.0)	5 (8.3)	0 (1.5)	0 (0.0)	9 (15.0)	2 (3.3)	2 (3.3)	0 (0.0)	5 (8.3)	60
2016	21 (30.9)	18 (26.5)	0 (0.0)	1 (1.5)	1 (1.5)	13 (19.1)	1 (1.5)	1 (1.5)	2 (2.9)	10 (14.7)	68
2017	10 (33.3)	5 (16.7)	4 (13.3)	0 (0.0)	0 (0.0)	5 (16.7)	1 (3.3)	0 (0.0)	1 (3.3)	4 (13.3)	30
2018	2 (20.0)	2 (20.0)	0 (0.0)	0 (0.0)	0 (0.0)	1 (10.0)	1 (10.0)	0 (0.0)	1 (10.0)	3 (30.0)	10

HTN, hypertension; ACEi, Angiotensin–converting enzyme inhibitors; ARB, angiotensin receptor blockers; BB, beta–blockers; CCB, calcium channel blockers; DU, diuretics; ECG, electrocardiogram.

The proportion of combination therapies increased when anti-HTN medications were changed, while the proportion of monotherapy decreased. The proportion of monotherapy reduced from 63.2 to 37.1% when anti-HTN medications were changed (Figure 2). On the other hand, the proportion of combination therapies increased from 36.6 to 62.9% when anti-HTN medications were changed.

For the second choice of anti-HTN medication, ARB+CCB combination was most frequent (54.3%), followed by ARB+DU combination (16.8%). Adding another class of anti-HTN medications that were not part of their previous regimens was observed most frequently when anti-HTN medications were changed (29.2%, Table 3).

**Table 3:** Number of changes in anti-hypertensive medications by class and mode of changes.

**Class of anti-hypertensive medications**	**Mode of changes in anti-hypertensive medications**						**Total changes**
	**Dose increased**	**Dose reduced**	**Medication added**	**Medication removed**	**Medication changed within the same class**	**Medication changed to a different class**	
CCB	6 (3.9)	4 (2.6)	101 (66.4)	0 (0.0)	14 (9.2)	27 (17.8)	152
ARB	23 (16.1)	13 (9.1)	46 (32.2)	0 (0.0)	31 (21.7)	30 (21.0)	143
BB	3 (10.0)	4 (13.3)	11 (36.7)	0 (0.0)	5 (16.7)	7 (23.3)	30
DU	1 (20.0)	0 (0.0)	3 (60.0)	0 (0.0)	0 (0.0)	1 (20.0)	5
ACEi	2 (50.0)	0 (0.0)	1 (25.0)	0 (0.0)	0 (0.0)	1 (25.0)	4
ARB, CCB	7 (7.1)	17 (17.2)	13 (13.1)	19 (19.2)	17 (17.2)	26 (26.3)	99
ARB, DU	4 (11.1)	0 (0.0)	8 (22.2)	12 (33.3)	2 (5.6)	10 (27.8)	36
ARB, BB	5 (14.3)	1 (2.9)	4 (11.4)	10 (28.6)	2 (5.7)	13 (37.1)	35
BB, CCB	2 (7.4)	2 (7.4)	1 (3.7)	7 (25.9)	5 (18.5)	10 (37.0)	27
CCB, DU	1 (10.0)	1 (10.0)	0 (0.0)	5 (50.0)	2 (20.0)	1 (10.0)	10
BB, DU	0 (0.0)	1 (14.3)	3 (42.9)	1 (14.3)	1 (14.3)	1 (14.3)	7
ACEi, BB	3 (60.0)	0 (0.0)	0 (0.0)	2 (40.0)	0 (0.0)	0 (0.0)	5
ARB, BB, CCB	2 (5.0)	4 (10.0)	2 (5.0)	15 (37.5)	7 (17.5)	10 (25.0)	40
ARB, CCB, DU	3 (9.4)	3 (9.4)	3 (9.4)	15 (46.9)	1 (3.1)	7 (21.9)	32
ARB, BB, CCB, DU	0 (0.0)	5 (26.3)	0 (0.0)	6 (31.6)	6 (31.6)	2 (10.5)	19
ARB, BB, DU	0 (0.0)	0 (0.0)	2 (20.0)	5 (50.0)	0 (0.0)	3 (30.0)	10
ACEi, BB, DU	0 (0.0)	0 (0.0)	0 (0.0)	3 (60.0)	0 (0.0)	2 (40.0)	5
Other combination therapies	1 (3.8)	1 (3.8)	2 (7.7)	15 (57.7)	0 (0.0)	7 (26.9)	26
Total	63 (9.2)	56 (8.2)	200 (29.2)	115 (16.8)	93 (13.6)	158 (23.1)	685

ACEi, Angiotensin-converting enzyme inhibitors; ARB, angiotensin receptor blockers; BB, beta-blockers; CCB, calcium channel blockers; DU, diuretics.

Proportion of changes in anti-hypertensive medications was calculated by dividing the number of changes by total number of changes for each drug(s).

### Factors associated with the changes in anti-hypertensive medications

Subjects with higher diastolic blood pressure were 1.02 times more likely to have their anti-HTN medications changed than those with lower diastolic blood pressure (95% confidence interval [CI]: 1.001–1.040, *p* = 0.049, Table 4). In addition, subjects with arrhythmia were 10.01 times (95% CI: 1.86–185.57, *p* = 0.030) more likely to have their anti-HTN medications changed than those without arrhythmia. Lastly, subjects with angina pectoris who also had received antianginal agents were 4.85 times (95% CI: 1.05–23.89, *p* = 0.046) more likely to have their anti-HTN medications changed than those without angina pectoris and antianginal agents.

**Table 4:** Results of univariate and multivariate analyses assessing the association between factors and changes in anti-hypertensive medications.

**Factors**	**Univariate analysis**		**Multivariate analysis**	
	**Crude OR (95% CI)**	***p*–value**	**Adjusted OR (95% CI)**	***p*–value**
Index year	0.95 (0.88–1.03)	0.244	–	–
**Demographics**				
Age	1.01 (0.99–1.03)	0.226	1.02 (0.99–1.03)	0.070
Male	1.15 (0.83–1.59)	0.409	1.36 (0.96–1.94)	0.087
**Hospitalization**	1.18 (0.79–1.76)	0.417	–	–
**Blood pressure**				
Systolic blood pressure	1.012 (1.004–1.021)	0.003	1.00 (0.99–1.02)	0.515
Diastolic blood pressure	1.02 (1.01–1.04)	0.001	1.020 (1.001–1.040)	0.049
**No. of comorbidities**	1.06 (0.96–1.18)	0.237	–	–
**No. of concomitant medications**	1.02 (0.92–1.12)	0.768	–	–
**Medical history: general**				
Diabetes mellitus	0.89 (0.60–1.31)	0.549	–	–
Diabetic neuropathy	0.66 (0.09–4.04)	0.656	–	–
Dyslipidemia	0.92 (0.67–1.27)	0.621	–	–
Eye diseases	0.81 (0.46–1.43)	0.472	–	–
Gastrointestinal diseases	1.28 (0.81–2.02)	0.297	–	–
Hyperuricemia	1.68 (0.41–8.24)	0.481	–	–
Hypothyroidism	1.68 (0.41–8.24)	0.481	–	–
Insomnia	0.33 (0.07–1.11)	0.096	0.30 (0.06–1.03)	0.074
Liver diseases	1.17 (0.72–1.92)	0.532	–	–
Mental and behavioral diseases	1.58 (0.68–3.86)	0.291	1.92 (0.81–4.79)	0.146
Musculoskeletal diseases	1.27 (0.69–2.37)	0.439	1.62 (0.85–3.14)	0.146
Neoplasm	1.00 (0.55–1.83)	1.000	–	–
Nervous system disorders	0.66 (0.09–4.04)	0.656	–	–
Obesity	0.66 (0.17–2.34)	0.526	–	–
Prediabetes	1.00 (0.40–2.47)	1.000	–	–
Prostatic hyperplasia	1.15 (0.41–3.31)	0.794	–	–
Pulmonary diseases	0.93 (0.44–1.97)	0.849	–	–
Renal diseases	0.89 (0.52–1.53)	0.681	–	–
Symptoms	1.26 (0.87–1.82)	0.225	–	–
**Medical history: cardiovascular disease**				
Abnormal ECG	1.10 (0.46–2.69)	0.824	–	–
Atrial fibrillation	1.87 (0.70–5.48)	0.225	–	–
Angina pectoris	1.69 (0.89–3.27)	0.113	0.91 (0.35–2.35)	0.846
Arrhythmia	10.31 (1.96–189.89)	0.027	10.01 (1.86–185.57)	0.030
Cerebrovascular diseases	0.97 (0.59–1.59)	0.899	–	–
Coronary arteriosclerosis	1.10 (0.60–2.02)	0.759	–	–
Heart failure	0.66 (0.17–2.34)	0.526	–	–
NSTEMI	2.01 (0.19–43.32)	0.570	–	–
**Medication use**				
Analgesic agents	1.32 (0.69–2.57)	0.085	–	–
Antianginal agents	1.14 (0.64–2.03)	0.661	0.75 (0.33–1.65)	0.484
Antianxiety agents	1.00 (0.40–2.47)	1.000	–	–
Antibacterials	1.00 (0.39–2.60)	1.000	–	–
Anticoagulant agents	1.32 (0.87–2.02)	0.199	–	–
Anticonvulsants	1.00 (0.23–4.27)	1.000	–	–
Antidepressants	1.68 (0.41–8.24)	0.481	–	–
Antidiabetic agents	0.88 (0.57–1.37)	0.576	–	–
Antigout agents	1.68 (0.41–8.24)	0.481	–	–
Gastrointestinal agents	1.09 (0.79–1.50)	0.622	–	–
HMG–CoA reductase inhibitors	0.78 (0.57–1.08)	0.139	0.77 (0.54–1.09)	0.134
Micturition disorder drugs	1.15 (0.41–3.31)	0.794	–	–
NSAIDs	0.97 (0.70–1.36)	0.865	–	–
Respiratory system agents	1.30 (0.69–2.49)	0.419	–	–
Sedatives	1.02 (0.71–1.47)	0.926	–	–
**Interaction**				
Angina pectoris: Antianginal agents	–	–	4.85 (1.05–23.89)	0.046

OR, odds ratio; CI, confidence interval; ECG, electrocardiogram; NSTEMI, non-ST-elevation myocardial infarction; NSAIDS, non-steroidal anti-inflammatory drugs.

The results of the multivariate logistic regression analyses, which were reported using crude and adjusted odds ratios of factors associated with the changes in anti–hypertensive medications and its 95% confidence intervals.

The logistic regression models included the main effects and 2nd order interactions between the main effects.

A sensitivity analysis by changing the period of baseline, i.e., 1 year to 6 months from the index date, did not practically modify the results of the main analysis. In other words, higher diastolic blood pressure and arrhythmia were consistently significantly associated with changes in anti-HTN medications and angina pectoris with antianginal agents was marginally significant for changes in anti-HTN medications (Supplementary Table 2).

## Discussion

We explored the pattern of anti-HTN medications and identified the factors associated with the medication changes in real world using a clinical database of a university-affiliated tertiary-care hospital. In our results, about two-thirds (63.2%) of subjects started treatments using one anti-HTN medication (Figure 2). In addition, subjects with higher diastolic blood pressure, arrhythmia, and angina pectoris with antianginal agents had their anti-HTN medications changed significantly more likely than those without (Table 4).

Our study results were consistent with those of a previous study in Korea using a claims database, which reported ARB (51.6%), CCB (45.0%), and BB (18.5%) were the most frequently used anti-HTN medications regardless of usage as mono- or combination-therapy (23). Similarly, most subjects received ARB (29.9%) as the initial treatment in our study, followed by CCB (26.3%), ARB+CCB (15.6%), and BB (5.7%, Table 2). In addition, a former study reported that ARB+CCB (12.59%) and ARB+DU (9.79%) were the most frequently used combination therapies, which was also comparable to our study results (15.6 and 4.4%, respectively, Table 2 and Figure 2) (23). Although a single clinical database was analyzed in our study, the patterns of anti-HTN medications were in line with those from other studies that used a nationwide database (23, 24).

For the initial monotherapy between 2010 and 2018, ARB and CCB were consistently highly used (i.e., ≥20%), whereas the uses of ACEi and DU were steadily low (i.e., ≤2%) (Table 2). A much smaller proportion of subjects received ACEi or DU, i.e., 0.8 and 0.5%, respectively, as the initial monotherapy (Table 2). We postulate this was mainly because ACEi or DU were not as effective as ARB or CCB to prevent adverse cardiovascular outcomes or to reduce elevated blood pressure. To support this notion, a previous retrospective observational study using the Korean National Health Insurance Service database showed that DU was inferior to ARB (hazard ratio [HR]: 0.42) but superior to ACEi (HR: 1.80) for preventing cardiovascular death, myocardial infarction, or stroke (25). Furthermore, we found that ≥75% of subjects either had their dose increased or had other medications added after initial treatments with ACEi or DU, which may indicate that initial choices of ACEi and DU were not as effective as expected to reduce elevated blood pressure (Table 3).

Multiple pathophysiological dysregulations involving the renin-angiotensin-system, autonomic nervous system, and endothelium contribute to the development of essential HTN (26). For this reason, the 2018 Korean Society of Hypertension Guideline have recommended to use combination therapies, which may act on multiple pathophysiological pathways, when initial monotherapy is not sufficient to reduce elevated blood pressure (20). This recommendation was added to the guideline based on a previous meta-analysis showing that the initial combination therapies exert two- to five-times greater anti-HTN effect and an additional 4% points reduction in the occurrence of coronary heart disease than those obtained by monotherapy (27, 28). Consistent with the recommendations provided by the Korean HTN guideline, the proportion of initial combination therapy have increased from 36.8 to 62.9% when initial anti-HTN medications were changed (Figure 2).

Changes in anti-HTN medications other than dose increase, dose decrease or removing ≥1 medication may represent a clinical setting where the previous treatment regimen was not adequate for the patient. Particularly, patients might have other medications added after previous treatments because the previous dosage regimen was not sufficient to reduce elevated blood pressure. Our results showed that 200 or 29.2% patients had other medications added after previous treatments to further reduce an elevated blood pressure, 162 (81.0%) of whom were treated with monotherapy before the addition (Table 3). This indicated that monotherapy was an unsuccessful treatment regimen in about a quarter (23.6%) of the patients, which made them to use combination therapy to further reduce elevated blood pressure.

Frequent changes in anti-HTN medications can result in various problems including reduced adherence to the prescribed drug, increased hospitalization risk, new adverse events, and concerns about the new treatment (7, 29, 30). In this regard, identifying factors associated with changes in anti-HTN medications may help clinicians to optimize treatment strategies for individual patients and to triage patients for ambulatory care visits, particularly in areas with large number of patients and limited resources. In this study, we found that elevated diastolic blood pressure, arrhythmia, and angina pectoris with antianginal agents were significantly associated with the changes in anti-HTN medications (Table 4). Those findings were not entirely unexpected given arrhythmia and angina pectoris are often a sign of hypertensive cardiovascular diseases, which warrants immediate medical attention (20). Changes in anti-HTN medications, i.e., dose increase, adding or changing ≥1 medications, that contain BB or CCB to treat arrhythmia and angina pectoris may have contributed to the frequent changes in anti-HTN medications (20, 31, 32).

The major strength of this study is its replicability, as we used OMOP-CDM for the study objectives (33). All databases converted to the OMOP-CDM can be used to explore the pattern of anti-HTN medications using the same set of queries, which can be downloaded from a Github webpage (https://github.com/chung7k/Treatment-pathway_anti-HTN-medication). Unlike other studies where only the use of monotherapies was explored, we also identified the use of combination therapies (15, 34).

This study had two major limitations. First, our study population might have included only adherent subjects because eligible patients had to have their prescriptions refilled continuously for 3 years. This might have resulted in a selection bias because factors associated with the changes in anti-HTN medications could have been different between patients who sticked to their prescribed medications and those who didn't. Nevertheless, selecting subjects with continuous treatment was necessary to ensure subjects did not receive anti-HTN medications from other medical institutions. Because the numbers of comorbid diseases and medications were significantly associated with poor medication adherence (35), further studies are warranted in patients with poor medication adherence. Second, although propensity score matching was performed between the changed and unchanged groups, which was successful in that all of the baseline characteristics between the groups became comparable (Table 1), there could have been some covariates that did not distribute evenly between the groups. This could have affected the way that a specific covariate was associated with changes in anti-HTN medications. To minimize this possibility, we adjusted for those covariates in multiple logistic regression analysis.

In conclusion, we successfully explored the pattern of anti-HTN medications and identified the factors associated with the medication changes. We found that clinical practices in SNUH was consistent with the clinical recommendations provided by the 2018 Korean Society of Hypertension Guideline (20). Subjects with high diastolic blood pressure, arrhythmia, and angina pectoris with antianginal agents were significantly associated with the changes in anti-HTN medications, indicating that any patients with these covariates require additional attention to reduce the likelihood of changing anti-HTN medications.

## Data availability statement

The original contributions presented in the study are included in the article/Supplementary material, further inquiries can be directed to the corresponding author/s.

## Ethics statement

The studies involving human participants were reviewed and approved by Seoul National University Hospital Institutional Review Board. Written informed consent for participation was not required for this study in accordance with the national legislation and the institutional requirements.

## Author contributions

TC, YJ, YH, SH, JM, and HL designed research and wrote the article. TC, SH, JM, and HL performed research. TC and HL analyzed data. All authors contributed to the article and approved the submitted version.

## Funding

This work was funded by Ministry of Health and Welfare (HI19C0572). This research was supported by the BK21FOUR Program of the National Research Foundation of Korea (NRF) funded by the Ministry of Education (5120200513755).

## Conflict of interest

The authors declare that the research was conducted in the absence of any commercial or financial relationships that could be construed as a potential conflict of interest.

## Publisher's note

All claims expressed in this article are solely those of the authors and do not necessarily represent those of their affiliated organizations, or those of the publisher, the editors and the reviewers. Any product that may be evaluated in this article, or claim that may be made by its manufacturer, is not guaranteed or endorsed by the publisher.
