# Supplementary material for: Factors affecting the changes in antihypertensive medications in patients with hypertension
Source: Front Cardiovasc Med. 2022 Sep 30;9:999548. doi: 10.3389/fcvm.2022.999548 (PMC9561640; doi:10.3389/fcvm.2022.999548)
Supplement: Supplementary file 1 [file Image_1.PDF]

Supplementary Material

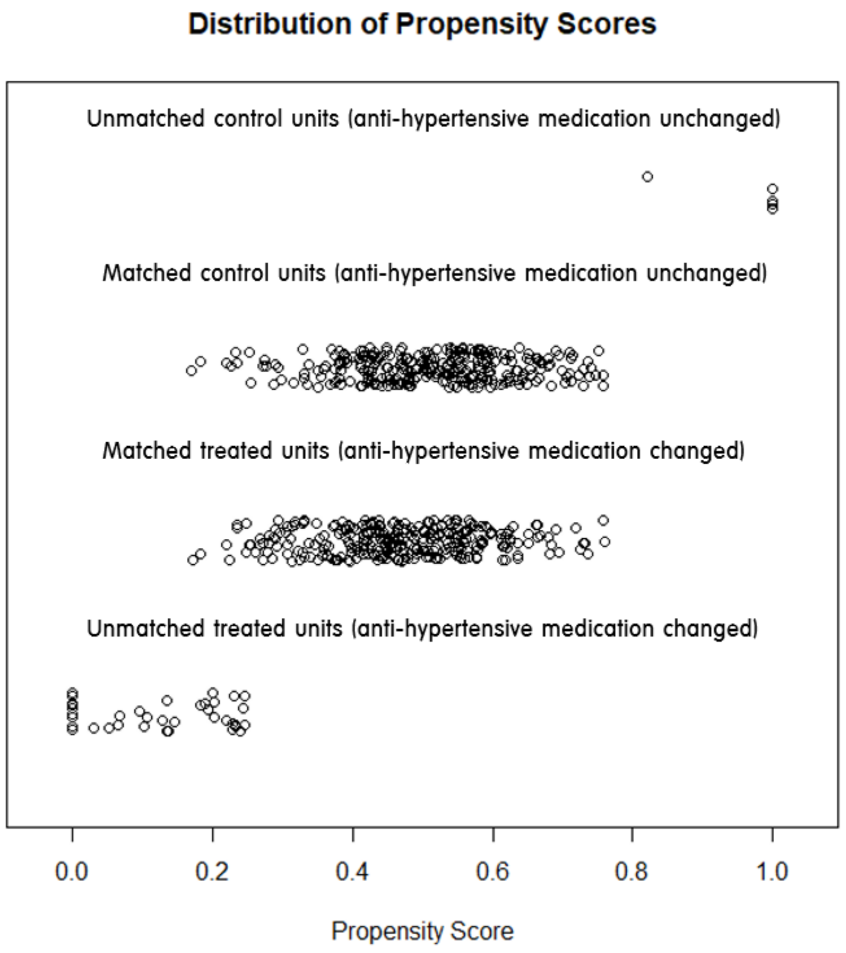

Supplementary Figure 1. Distribution of propensity scores using jitter plot.
