# Supplementary material for: Factors affecting the changes in antihypertensive medications in patients with hypertension
Source: Front Cardiovasc Med. 2022 Sep 30;9:999548. doi: 10.3389/fcvm.2022.999548 (PMC9561640; doi:10.3389/fcvm.2022.999548)
Supplement: Supplementary file 3 [file Table_1.DOCX]

Supplementary Material

Supplementary Table 1. Characteristics of patients with hypertension

| Factors | ***Between index date and cohort exit date*** | | |
| --- | --- | --- | --- |
|  | Total  (n = 594) | Anti-HTN  medication  changed  (n =297) | Anti-HTN  medication  unchanged  (n = 297) |
| **Index year,** year±SD | 2012.6±2.0 | 2012.5±2.0 | 2012.8±2.0 |
| **Demographics** |  |  |  |
| Male, n (%) | 353 (55.5) | 192 (57.5) | 161 (53.3) |
| **Medical history: general,** n (%) |  |  |  |
| Diabetes mellitus | 221 (37.2) | 108 (36.4) | 113 (38.0) |
| Diabetic neuropathy | 15 (2.5) | 5 (1.7) | 10 (3.4) |
| Dyslipidemia | 496 (83.5) | 244 (82.2) | 252 (84.8) |
| Eye diseases | 141 (23.7) | 67 (22.6) | 74 (24.9) |
| Gastrointestinal diseases | 249 (41.9) | 121 (40.7) | 128 (43.1) |
| Hyperuricemia | 20 (3.4) | 9 (3.0) | 11 (3.7) |
| Hypothyroidism | 15 (2.5) | 7 (2.4) | 8 (2.7) |
| Insomnia | 61 (10.3) | 31 (10.4) | 30 (10.1) |
| Liver diseases | 155 (26.1) | 76 (25.6) | 79 (26.6) |
| Mental and behavioral diseases | 83 (14.0) | 35 (11.8) | 48 (16.2) |
| Musculoskeletal diseases | 138 (23.2) | 70 (23.6) | 68 (22.9) |
| Neoplasm | 66 (11.1) | 32 (10.8) | 34 (11.4) |
| Nervous system disorders | 20 (3.4) | 9 (3.0) | 11 (3.7) |
| Obesity | 16 (2.7) | 8 (2.7) | 8 (2.7) |
| Prediabetes | 117 (19.7) | 61 (20.5) | 56 (18.9) |
| Prostatic hyperplasia | 79 (13.3) | 34 (11.4) | 45 (15.2) |
| Pulmonary diseases | 89 (15.0) | 52 (17.5) | 37 (12.5) |
| Renal diseases | 178 (30.0) | 78 (26.3) | 100 (33.7) |
| Symptoms | 331 (55.7) | 162 (54.5) | 169 (56.9) |
| **Medical history: cardiovascular disease** |  |  |  |
| Abnormal ECG | 30 (5.1) | 14 (4.7) | 16 (5.4) |
| Atrial fibrillation | 38 (6.4) | 14 (4.7) | 24 (8.1) |
| Angina pectoris | 78 (13.1) | 33 (11.1) | 45 (15.2) |
| Arrhythmia | 21 (3.5) | 5 (1.7) | 16 (5.4) |
| Cerebrovascular diseases | 141 (23.7) | 70 (23.6) | 71 (23.9) |
| Coronary arteriosclerosis | 87 (14.6) | 47 (15.8) | 40 (13.5) |
| Heart failure | 43 (7.2) | 21 (7.1) | 22 (7.4) |
| NSTEMI | 5 (0.8) | 2 (0.7) | 3 (1.0) |
| **Medication use**, n (%) |  |  |  |
| Analgesic agents | 241 (40.6) | 113 (38.0) | 128 (43.1)) |
| Antianginal agents | 165 (27.8) | 79 (26.6) | 86 (29.0) |
| Antianxiety agents | 85 (14.3) | 39 (13.1) | 46 (15.5) |
| Antibacterials | 132 (22.2) | 57 (19.2) | 75 (25.3) |
| Anticoagulant agents | 180 (30.3) | 83 (27.9) | 97 (32.7) |
| Anticonvulsants | 53 (8.9) | 26 (8.8) | 27 (9.1) |
| Antidepressants | 61 (10.3) | 23 (7.7) | 38 (12.8) |
| Antidiabetic agents | 162 (27.3) | 80 (26.9) | 82 (27.6) |
| Antigout agents | 24 (4.0) | 6 (2.0) | 18 (6.1) |
| Gastrointestinal agents | 510 (85.9) | 248 (83.5) | 262 (88.2) |
| HMG-CoA reductase inhibitors | 427 (71.9) | 217 (73.1) | 210 (70.7) |
| Micturition disorder drugs | 56 (9.4) | 28 (9.4) | 28 (9.4) |
| NSAIDs | 394 (66.3) | 200 (67.3) | 194 (65.3) |
| Respiratory system agents | 211 (35.5) | 104 (35.0) | 107 (36.0) |
| Sedatives | 343 (57.7) | 167 (56.2) | 176 (59.3) |
| SD, standard deviation; ECG, electrocardiogram; NSTEMI, non-ST-elevation myocardial infarction; NSAIDS, nonsteroidal anti-inflammatory drugs | | | |
