# Supplementary material for: Factors affecting the changes in antihypertensive medications in patients with hypertension
Source: Front Cardiovasc Med. 2022 Sep 30;9:999548. doi: 10.3389/fcvm.2022.999548 (PMC9561640; doi:10.3389/fcvm.2022.999548)
Supplement: Supplementary file 4 [file Table_2.DOCX]

Supplementary Material

Supplementary Table 2. Results of sensitivity analysis

| Factors | Univariate analysis | | Multivariate analysis | |
| --- | --- | --- | --- | --- |
|  | Crude OR  (95 % CI) | p-value | Adjusted OR  (95 % CI) | p-value |
| **Index year** | 0.95 (0.88-1.03) | 0.244 | - | - |
| **Demographics** |  |  |  |  |
| Age | 1.01 (0.99-1.03) | 0.226 | 1.02 (0.99-1.03) | 0.078 |
| Male | 1.15 (0.83-1.59) | 0.409 | 1.35 (0.95-1.92) | 0.097 |
| **Blood pressure** |  |  |  |  |
| Systolic blood pressure | 1.01 (1.004-1.021) | 0.003 | 1.00 (0.99-1.02) | 0.506 |
| Diastolic blood pressure | 1.02 (1.01-1.04) | 0.001 | 1.02 (1.001-1.040) | 0.044 |
| **Medical history: general** |  |  |  |  |
| Diabetes mellitus | 0.91 (0.61-1.34) | 0.617 | - | - |
| Diabetic neuropathy | 0.66 (0.09-4.04) | 0.656 |  |  |
| Dyslipidemia | 0.91 (0.66-1.26) | 0.562 | - | - |
| Eye diseases | 0.82 (0.44-1.52) | 0.531 | - | - |
| Gastrointestinal diseases | 1.06 (0.65-1.74) | 0.804 | - | - |
| Hyperuricemia | 1.34 (0.29-6.84) | 0.705 | - | - |
| Hypothyroidism | 1.34 (0.29-6.84) | 0.705 | - | - |
| Insomnia | 0.28 (0.04-1.17) | 0.115 | 0.24 (0.04-1.05) | 0.086 |
| Liver diseases | 1.22 (0.74-2.04) | 0.440 | - | - |
| Mental and behavioral diseases | 1.47 (0.66-3.60) | 0.387 | 1.72 (0.71-4.32) | 0.232 |
| Musculoskeletal diseases | 1.36 (0.73-2.60) | 0.338 | 1.68 (0.87-3.32) | 0.128 |
| Neoplasm | 0.89 (0.46-1.73) | 0.737 | - | - |
| Nervous system disorders | 0.66 (0.09-4.04) | 0.656 | - | - |
| Obesity | 0.60 (0.12-2.45) | 0.481 | - | - |
| Prediabetes | 1.00 (0.39-2.60) | 1.000 | - | - |
| Prostatic hyperplasia | 0.85 (0.27-2.60) | 0.779 | - | - |
| Pulmonary diseases | 0.85 (0.38-1.88) | 0.689 | - | - |
| Renal diseases | 1.20 (0.66-2.18) | 0.547 | - | - |
| Symptoms | 1.12 (0.44-2.85) | 0.816 | - | - |
| **Medical history: cardiovascular disease** |  |  |  |  |
| Abnormal ECG | 1.10 (0.44-2.85) | 0.816 | - | - |
| Atrial fibrillation | 1.87 (0.70-5.48) | 0.225 | - | - |
| Angina pectoris | 1.69 (0.89-3.27) | 0.113 | 0.98 (0.39-2.50) | 0.971 |
| Arrhythmia | 10.31 (1.96-189.89) | 0.027 | 10.05 (1.87-186.44) | 0.029 |
| Cerebrovascular diseases | 1.10 (0.60-2.05) | 0.754 | - | - |
| Coronary arteriosclerosis | 1.10 (0.60-2.05) | 0.754 | - | - |
| Heart failure | 0.66 (0.17-2.34) | 0.526 | - | - |
| NSTEMI | 2.01 (0.19-43.32) | 0.570 |  |  |
| **Medication use** |  |  |  |  |
| Analgesic agents | 1.96 (0.92-4.46) | 0.092 | - | - |
| Antianginal agents | 1.20 (0.66-2.18) | 0.547 | 0.85 (0.37-1.89) | 0.685 |
| Antianxiety agents | 1.00 (0.39-2.60) | 1.000 | - | - |
| Antibacterials | 1.17 (0.38-3.68) | 0.779 | - | - |
| Anticoagulant agents | 1.37 (0.89-2.11) | 0.157 | - | - |
| Anticonvulsants | 1.34 (0.29-6.84) | 0.705 | - | - |
| Antidepressants | 1.68 (0.41-8.24) | 0.481 | - | - |
| Antidiabetic agents | 0.83 (0.53-1.30) | 0.426 | - | - |
| Antigout agents | 2.53 (0.54-17.73) | 0.270 | - | - |
| Gastrointestinal agents | 0.97 (0.70-1.35) | 0.866 | - | - |
| HMG-CoA reductase inhibitors | 0.82 (0.59-1.13) | 0.217 | 0.81 (0.57-1.14) | 0.223 |
| Micturition disorder drugs | 0.85 (0.27-2.60) | 0.779 | - | - |
| NSAIDs | 0.94 (0.67-1.32) | 0.732 | - | - |
| Respiratory system agents | 1.33 (0.64-2.83) | 0.455 | - | - |
| Sedatives | 0.94 (0.63-1.40) | 0.761 | - | - |
| **Interaction** |  |  |  |  |
| Angina pectoris : Antianginal agents | - | - | 3.87 (0.84-19.08) | 0.088 |
| OR, odds ratio; CI, confidence interval; ECG, electrocardiogram; NSTEMI, non-ST-elevation myocardial infarction; NSAIDS, nonsteroidal anti-inflammatory drugs  The results of the multivariate logistic regression analyses, which were reported using crude and adjusted odds ratios of factors associated with the changes in anti-hypertensive medications and its 95 % confidence intervals.  The logistic regression models included the main effects and 2nd order interactions between the main effects. | | | | |
